# The scientific term paper at the Charité: a project report on concept, implementation, and students' evaluation and learning

**DOI:** 10.3205/zma001261

**Published:** 2019-10-15

**Authors:** Simon Drees, Florian Schmitzberger, Günter Grohmann, Harm Peters

**Affiliations:** 1 Charité – Universitätsmedizin Berlin, Freie und Humboldt-Universität zu Berlin, Prodekanat für Studium und Lehre, Dieter Scheffner Fachzentrum für Medizinische Hochschullehre und Ausbildungsforschung, Berlin, Germany

**Keywords:** scientific work, undergraduate medical education, curriculum development, term paper, scientific project, scientific competence, modular curriculum of medicine

## Abstract

**Aim: **Better training in scientific skills, such as the ability to conduct research independently, has been one of the main drivers of reform in medical education. The aim of this article is to report on the scientific term paper module in the modular curriculum of medicine (MCM) at the Charité. This module is an established example of undergraduate medical students conducting their own scientific investigations.

**Project outline: **A faculty-wide, outcome-oriented process resulted in a four-week module for writing a scientific term paper in the 6^th^ semester of the MCM as part of a longitudinal science curriculum. Acquired competencies were assessed through a written term paper and an oral presentation. Two student cohorts (winter terms 2013 and 2014) were surveyed on how they rated the module concept, organizational aspects and the quality of support. We further analysed the chosen topics of the papers as well as student assessment results.

**Results: **The student evaluation (return rates of 193 and 197, 71% and 77%) showed high overall satisfaction with the module. This result was evident in the high rating of the module concept and organizational aspects, a positive attitude towards scientific research, and strong motivation to pursue further scientific research. There was a wide spectrum of term paper topics with a focus on literature reviews. Most of the student work was assessed as good or very good.

**Conclusion: **The scientific term paper module has proven itself as a curricular concept for students to perform own scientific research in the MCM, with strong acceptance and good performance by students. This project report can serve as basis and guidance for development and further improvements to promote scientific competencies in undergraduate medical education in other faculties.

## Zusammenfassung

**Zielsetzung: **Die bessere Vermittlung wissenschaftlicher Kompetenzen, darunter die Fähigkeit zur eigenständigen wissenschaftlichen Arbeit, bildet eine der Haupttriebfedern für die Reform des Medizinstudiums. Ziel dieses Projektberichtes ist es, die wissenschaftliche Hausarbeit im Modellstudiengang Medizin (MSM) der Charité vorzustellen. Sie ist ein etabliertes Praxisbeispiel von eigener wissenschaftlicher Arbeit im Medizinstudium.

**Projektbeschreibung:** In einem fakultätsweiten, outcome-orientierten Prozess wurde ein 4-wöchiges Modul zur Erstellung einer wissenschaftlichen Hausarbeit im 6. Semester des MSM als Teil eines longitudinalen Wissenschaftscurriculums entwickelt und implementiert. Der Kompetenzerwerb wird auf Basis einer schriftlichen Hausarbeit und einer mündlichen Präsentation geprüft. In zwei Studierendenkohorten im Wintersemester 2013 und 2014 wurden Fragen zum Konzept des Moduls, zu organisatorischen Aspekten und zur Betreuungsqualität evaluiert. Außerdem wurden Statistiken zu den bearbeiteten Themen erhoben und die Ergebnisse der Leistungsbewertungen ausgewertet.

**Ergebnisse: **Die Studierendenevaluation (Rücklaufquote 193 bzw. 197 Studierende; 71% bzw. 77%) zeigte eine hohe Gesamtzufriedenheit auf. Diese manifestierte sich in einer guten Bewertung des Modulkonzeptes und der organisatorischen Aspekte sowie einer sehr positiven Einstellung zum Thema wissenschaftliches Arbeiten und einer hohen Motivation für eine weitere wissenschaftliche Arbeit. Die Themen der Hausarbeit waren weit gestreut mit Schwerpunkt auf Literaturarbeiten. Die Leistungen der Studierenden wurden zum Großteil als gut oder sehr gut bewertet.

**Schlussfolgerung: **Die wissenschaftliche Hausarbeit hat sich als curriculares Konzept für die Umsetzung von eigener wissenschaftlicher Arbeit im MSM mit hoher Akzeptanz und guter Leistung seitens der Studierenden bewährt. Dieser Projektbericht kann anderen Fakultäten als Grundlage und Orientierung für Entwicklung bzw. Weiterentwicklung eigener Programme zur Förderung von Wissenschaftskompetenz im Medizinstudium dienen.

## 1. Introduction

Better training in scientific thinking and practice has, along with the acquisition of clinical-practical and communication skills, been one of the main drivers in the reform of undergraduate medical education that began two decades ago in Germany. Curricular frameworks such as the National Competency-based Catalogue of Learning Objectives for Medicine (NKLM) include scientific practice and the critical appraisal of scientific results as core competencies and outcomes of undergraduate medical education [http://www.nklm.de], [1], [2], [3], [4]. The model clause and reformed regular medical programmes offer new opportunities to implement the teaching, learning and assessing of students’ own scientific practice. A challenge for faculties is to reconcile formal, curricular and organizational parameters with the relatively early-stage development of students’ scientific competencies. The published literature on this topic lacks practice examples to provide the basis and orientation for developing and implementing these programmes. This project report therefore aims to describe the concept and structure of the “scientific term paper” of the model clause-type modular curriculum of medicine at Charité – Universitätsmedizin Berlin (Charité) and report on its implementation based on student evaluation data and assessment results. 

In German medical faculties, scientific competencies are often taught implicitly and unconnectedly across teaching formats and semesters [4] rather than explicitly as a longitudinal curriculum with defined outcomes. A lack of scientific competencies among medical students and graduates has been revealed by several studies [3], [5], [6], [7]. Important stakeholder groups such as the Science Council (Wissenschaftsrat), the German Research Foundation and the German Medical Students’ Association have demanded the strengthening of scientific training in medical education in Germany [3], [8], [9]. The NKLM, based on the Royal College of Surgeons and Physicians of Canada CanMEDS framework, defines the medical role of the scholar and the corresponding outcomes and learning objectives for undergraduate medical education in Germany [3], [10]. In addition to the critical appraisal of information sources and the use of evidence-based medicine in clinical practice, undergraduate medical education should enable students to conduct their own scientific research. The curricular implementation of this training outcome can be achieved in both regular and model clause medical programmes. This entails considering general context features, such as the discipline-focused requirements of medical licensure laws and the existing degree of curricular integration as well as existing teaching traditions and experiences with curricular innovation at the respective faculty [11].

Several German faculties have recently begun the development and implementation of teaching formats and modules for scientific training in their medical degree programmes [3], [4], [12], [13], [14], [15], [16], [17], [18], [19], [20], [21], [22], [23], [24]. Nevertheless, the literature lacks examples of practice in terms of how curricular implementation can be achieved in the German national context. The Charité has many years of experience with the concept of a “scientific term paper” conducted by students with the support and supervision of a scientist [25], [26]. It is referred to as a “term paper” because students perform most of the work at home outside of regular learning activities. The scientific term paper was already a component of the discipline-based regular curriculum of medicine (7^th^ semester, since 2003) as well as the reformed curriculum of medicine at the Charité (4^th^ semester, since 2001). Teaching sessions to prepare and support students in their own scientific work were distributed across several semesters in the regular curriculum, while they were carried out in one block in the reformed curriculum. In the model clause modular curriculum of medicine (MCM), which was introduced at the Charité in 2010, a dedicated module for scientific training was developed, and we can now report on the practical experience of this training.

This project report aims to describe the concept and implementation of the “scientific term paper” module in the modular curriculum of medicine at Charité, together with its effectiveness based on student evaluation data and assessment results.

## 2. Project outline

### 2.1. Setting

The scientific term paper module occurs in the 6^th^ semester of the modular curriculum of medicine at the Charité, which was introduced in 2010. Years 1-5 are structured as 40 themed, integrated modules [11]. The curriculum is competency-based, and the curriculum planning is outcome-oriented. “Scientific thinking and working” is one of the overarching competency areas [27].

#### 2.2. Design and structure

The module design followed a two-step process in which the whole faculty, including students, was involved.

##### 2.2.1. Phase 1: Blueprint planning

The overarching blueprint planning took place on an interdisciplinary committee commissioned by the faculty council for planning the modular curriculum of medicine (KEMM). A learning spiral consisting of 3 coordinated modules was decided upon: introduction to scientific practice (including a one-week guided “small scientific project”), scientific term paper (own research project, the subject of this article), and scientific practice in daily clinical practice (no project work). For each module, the KEMM committee determined the overarching module outcomes, the scope and type of teaching formats, group sizes and the type of assessment. Table 1 (Tab. 1) gives an overview of the module order, respective semester and module outcomes as documented in the study regulation. The planning by the KEMM committee was based on previous experience with scientific work in regular and reformed degree programmes, consultation with curriculum developers at the Charité and the corresponding published literature.

The module outcomes for module 23 on the scientific term paper replicate the research process, from the development of a research question to the presentation and discussion of results, in consideration of scientific and ethical standards (see table 1 (Tab. 1)). These are aligned with the NKLM (chapters 6.4 and 14a.3) [http://www.nklm.de]. The module is four weeks long with 72 teaching units in various formats. The submitted term paper and an oral presentation constitute the assessment for this module.

##### 2.2.2. Phase 2: Detailed planning

In 2012, the content and structure of the module “Scientific thinking and working II” was planned in an 8-step process led by the project steering group for the MCM together with representatives of the 34 departments at the Charité. Table 2 (Tab. 2) gives an overview of the course topics and formats. Most consist of supervised scientific research to give students sufficient time for their own work on their scientific term paper. Altogether, 6 teaching units in group sizes of 16 are planned for the oral presentation of the scientific term paper. All scientific staff at the Charité are permitted to supervise scientific term paper projects. The supervisors’ activity is credited as a regular teaching service and thereby also taken into account for their habilitation. A further incentive is the potential to recruit doctoral students. The recruitment of supervisors is facilitated further by the size of the faculty, the longstanding tradition with this concept and the participatory curriculum development process. The students may use the next module (M24, “elective I”, four weeks) to further engage with the topic of their scientific term paper. Students must indicate their wish to do this at the beginning of module 23.

A criteria-based grading sheet was prepared for the assessment of the written term paper and the oral presentation. The content of the written scientific paper is assessed by the supervisor, and the structure is assessed by the office for “scientific term papers”. The oral presentation consists of a short presentation and a question and answer session. This is assessed by two examiners (scientific staff from different departments within the Charité), reflecting both the presentation and the answers to questions.

The results of the module planning process were reviewed by the study committee and the assessment board and were approved following minimal modifications.

#### 2.3. Implementation

The module “Scientific thinking and working II” was carried out for the first time in the summer semester of 2013. The students were informed about the module via an internal online platform. The office for “scientific term papers” within the Dean´s Office for Student Affairs provided organizational and content-related support. Students were able to select a topic and a supervisor for their scientific term paper independently. This occurred either via direct contact, such as via e-mail, or via an online database of topics established specifically for this purpose (the scientific module database “Minerva”). Scientific staff were able to enter topic suggestions into this database, which could be selected by the students or used as a starting point for developing a topic together. Such a shared topic development and direct contact could build on the focus of the respective working group and take into account the particular methodological or thematic interests of the students in the design of the topic. A student who did not select a topic was assigned a topic and a supervisor from the online pool.

#### 2.4. Student evaluation and learning success

Two evaluations were carried out, one each in the winter semesters of 2013 and 2014. Basic data on the number of students and selected topics were drawn from the internal university information system and the module database “Minerva”. The evaluation was conducted using an anonymous, paper-based questionnaire that was distributed to the students when turning in their scientific term papers. These surveys were independent of the general evaluation of the module. The questionnaires contained 3-, 4-, 5-, and 6-point Likert scales and dichotomous answer options.

In the first survey in the winter semester of 2013 (second run of the module), the students were surveyed on the quality of the supporting infrastructure, workload, motivation and acceptance of scientific work and the overall concept of the module. In the second survey in the winter semester of 2014 (fourth run of the module), the focus of the survey was on cooperation with the supervisor and a self-assessment of learning success.

In addition to these surveys, data from subsequent evaluations of the module in the winter semesters of 2016 and 2017 and the summer semester of 2018 regarding overall student satisfaction with the module as well as satisfaction with learning gain were analysed (with 5-point Likert scales). Additionally, dichotomous answer options were used to assess the degree to which the minimum criteria for good support and supervision developed as a result of the second survey were guaranteed.

In addition, the assessment results for both the written term paper and the presentation in the winter semester of 2014 were analysed. The assessment of the scientific term paper consisted of two parts:

1. Grading of formal aspects by the “scientific term paper” office team and
2. Grading of the content by the supervisor.

The oral presentation is assessed by two scientific staff members and covers both the content and formal aspects of the presentation and discussion. This assessment is based on a checklist with several subcategories for which points are awarded and finally translated into a school grade (see the attachment 1 ). These checklists are accessible to students.

#### 2.5. Analysis

The quantitative analysis of the data was conducted descriptively using SPSS (Version 25).

## 3. Results

### 3.1. Basic information on the number of students and scientific paper topics

During the first evaluation, 297 students produced a scientific term paper. Of these, 243 selected a topic themselves (82%), and 44 students (15%) were assigned a topic from the Minerva pool. During the second evaluation, 280 students produced a scientific term paper, with 85% choosing their own topic and 15% being assigned a topic. Twenty-one percent of students from the first evaluation and 31% from the second evaluation chose to further investigate their selected topic for the scientific paper as part of their elective module. Table 3 (Tab. 3) gives an overview of the topic distribution for scientific papers in both semesters. The most commonly selected types of topics are literature reviews, clinical studies and laboratory work.

A total of 193 students took part in the first evaluation, and 197 participated in the second evaluation. These numbers correspond to 71% and 77% of the semester cohort, respectively. In the following, the results of the student evaluation and assessment are presented grouped by theme.

#### 3.2. Supporting infrastructure, workload and required time (1st survey)

**Figure 1:**
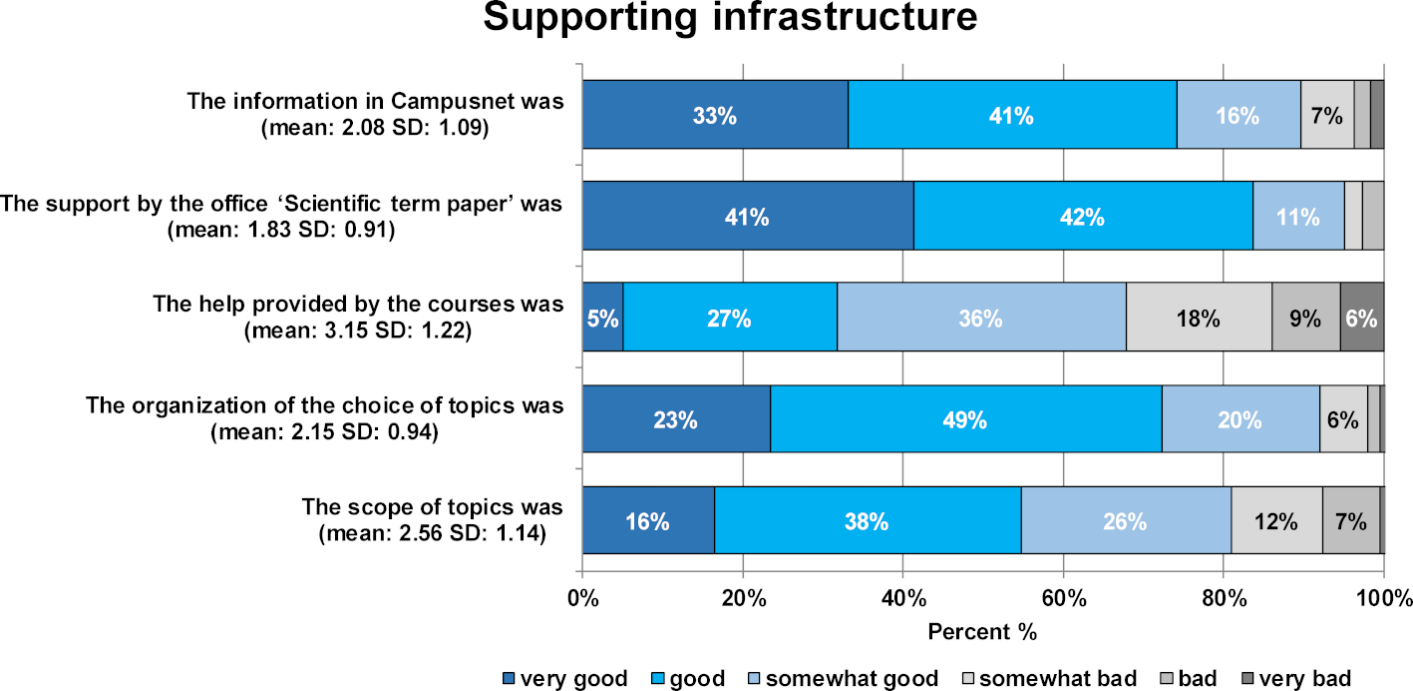
Results of the student evaluation of supporting infrastructure during the preparation of the scientific term paper (1^st^ survey). In each case, the percentage of students who rated the supporting infrastructure on a 6-point Likert scale from "very good" to "very bad" is shown. The mean and standard deviation are given in brackets.

The infrastructure for producing the scientific term paper was rated as good by the majority of students. Figure 1 (Fig. 1) shows students' ratings of the support offered by the faculty, the courses and the attractiveness and variety of available topics. The students reported a mean estimated required time of 80 hours of work (n=141, range: 8-300, MW: 99.16, SD: 60.51). The mean estimated time required by the supervisor was 5 hours (n=162, range: 0.5-100, MW: 11.37, SD: 16.72). The motivation for scientific research before and after producing the paper was positive at 86% and 87%, respectively. The written term paper and presentation exam formats were rated as sensible by 94% and 98% of the students, respectively.

#### 3.3. Cooperation with supervisor (1st and 2nd surveys)

**Figure 2:**
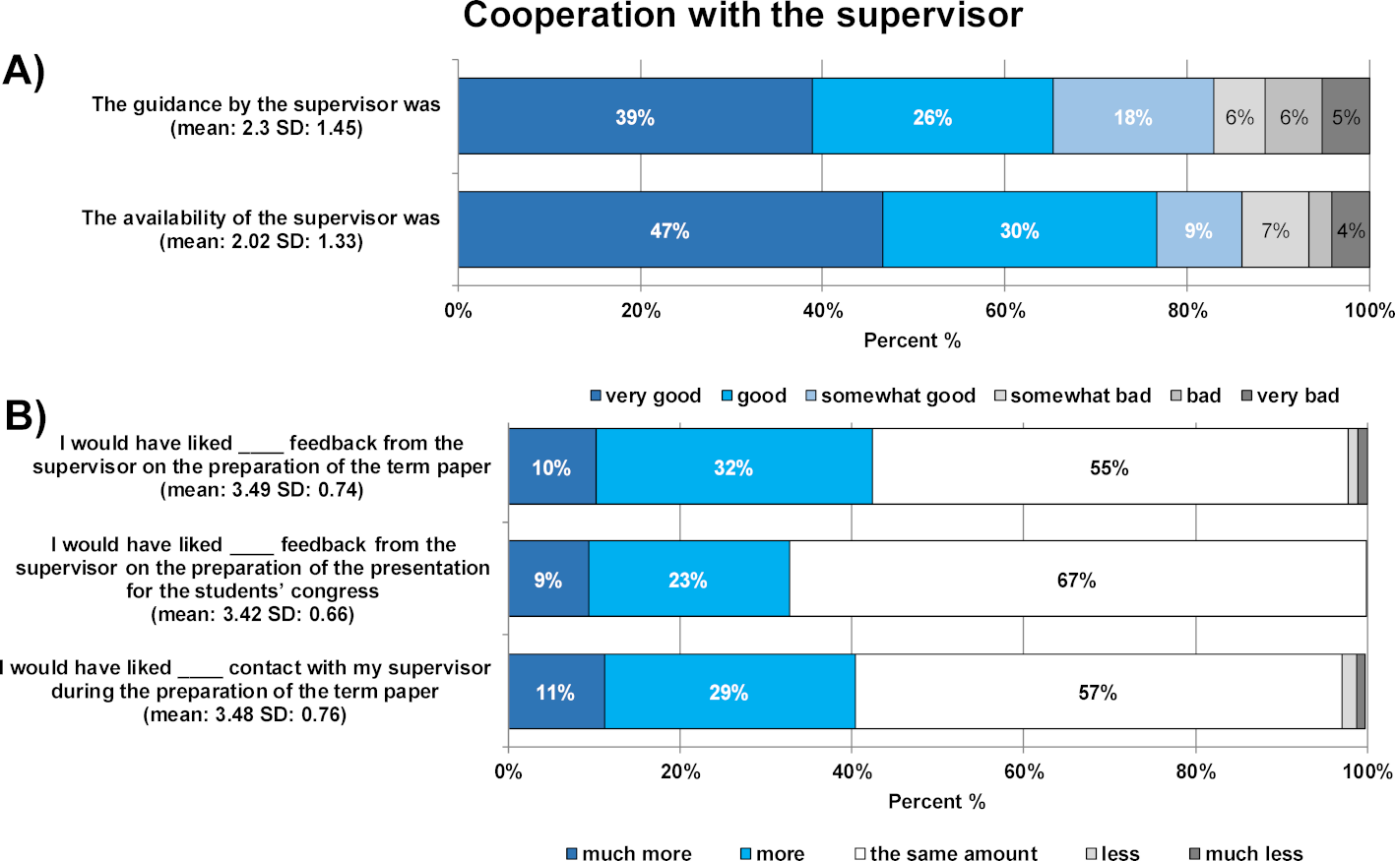
Results of the student evaluation of cooperation with the supervisor during the preparation of the scientific term paper. Part A) shows the percentage of students who rated areas of cooperation on a 6-point Likert scale from "very good" to "very bad" (1^st^ survey). Part B) shows the percentage of students who rated the level of feedback and contact on a 5-point Likert scale from "much more" to "much less" (2^nd^ survey). The mean and standard deviation are given in brackets.

The majority of students reported that they had met their supervisor one to four times and had email or telephone contact three to seven times or even more (52.9% and 73.3%). Furthermore, 83% of students stated that they received a quick response to their questions. Figure 2 (Fig. 2), part a (1^st^ survey) shows how the overwhelming majority of students rated instruction from their supervisor and their availability as good. Figure 2 (Fig. 2), part b (2^nd^ survey) shows that the majority of students preferred to have the same amount of feedback on their scientific term paper (55%) and on their presentation for the students’ congress (67%) as well as the same amount of contact with their supervisor (55%); 42%, 31% and 40% of students, respectively, would have preferred more contact. The 2^nd^ survey showed that a small percentage of students prepared for the oral presentation together with their supervisor: 62% discussed the presentation beforehand, 17% undertook a trial run in their department, and 38% did not discuss their presentation beforehand.

The share of students who had begun a doctoral project before they worked on their scientific term paper was 14%. After their scientific term paper, 27% of students planned to start a doctoral project with their supervisor, and 20% considered it. Altogether, 85% of students recommended their supervisor for future scientific term paper projects.

#### 3.4. Learning success (1st survey)

**Figure 3:**
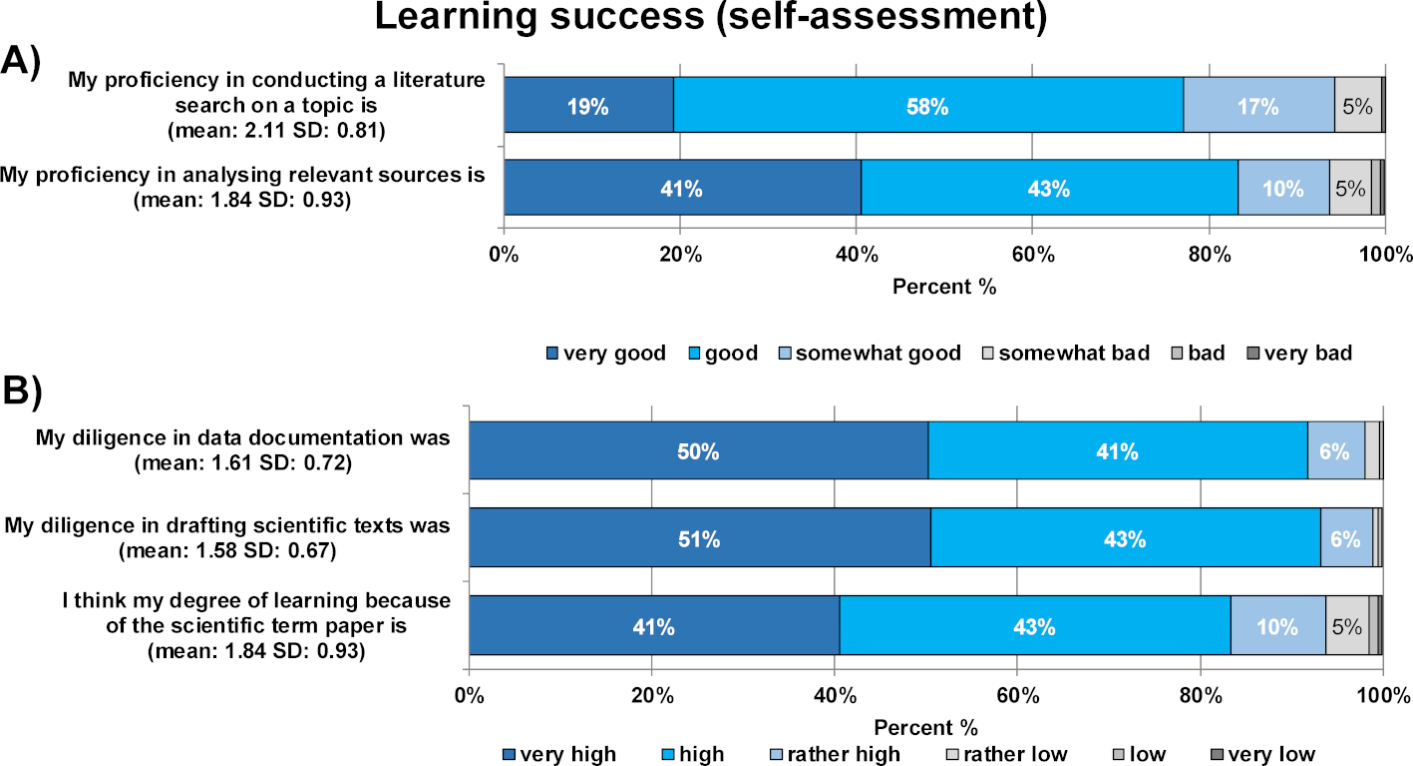
Results for the student evaluation of learning success in module 23 "Scientific thinking and working II" (1^st^ survey). Part A) shows the percentage of students who rated their acquired scientific skills on a 6-point Likert scale from "very good" to "very bad". Part B) shows the percentage of students who rated their diligence in scientific work and learning growth on a 6-point Likert scale from "very high" to "very low". The mean and standard deviation are given in brackets.

Students’ self-evaluation of the skills needed for their own scientific research acquired through the module is illustrated in figure 3 (Fig. 3). The majority of students reported having a high degree of diligence in data documentation and scientific writing. Additionally, they showed high self-confidence in both assessing sources and conducting literature research, and 84% of students rated their individual learning growth in module 23 as very good or good.

**Figure 4:**
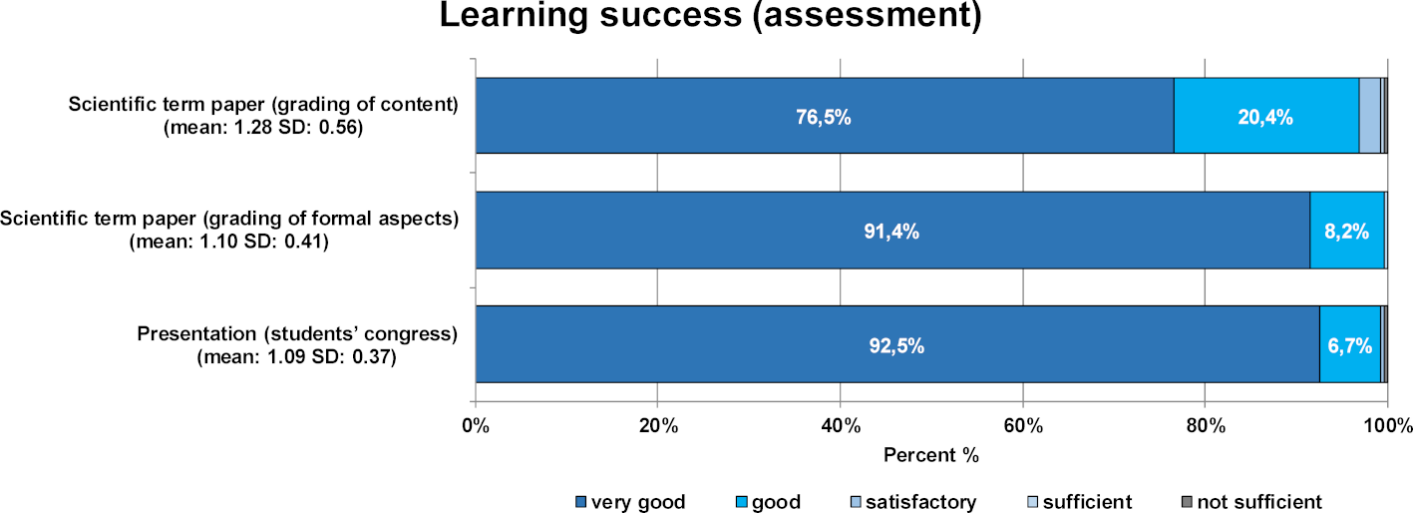
Results for learning success based on the assessment for module 23 “Scientific practice II”, showing the distribution of grades from “very good” to “not sufficient” for the written paper (content and formal grading) and the oral presentation at the students’ congress. The mean and standard deviation are given in brackets.

Students’ self-evaluations are congruous with the assessment results (see figure 4 (Fig. 4)). For the written term paper, 96% and 99.6% of students achieved the grade “very good” and “good” in the grading of content and formal aspects, respectively. A total of 99.2% of the oral presentations at the students’ congress were graded as “very good” and “good”.

#### 3.5. Acceptance of scientific research (2nd survey)

**Figure 5:**
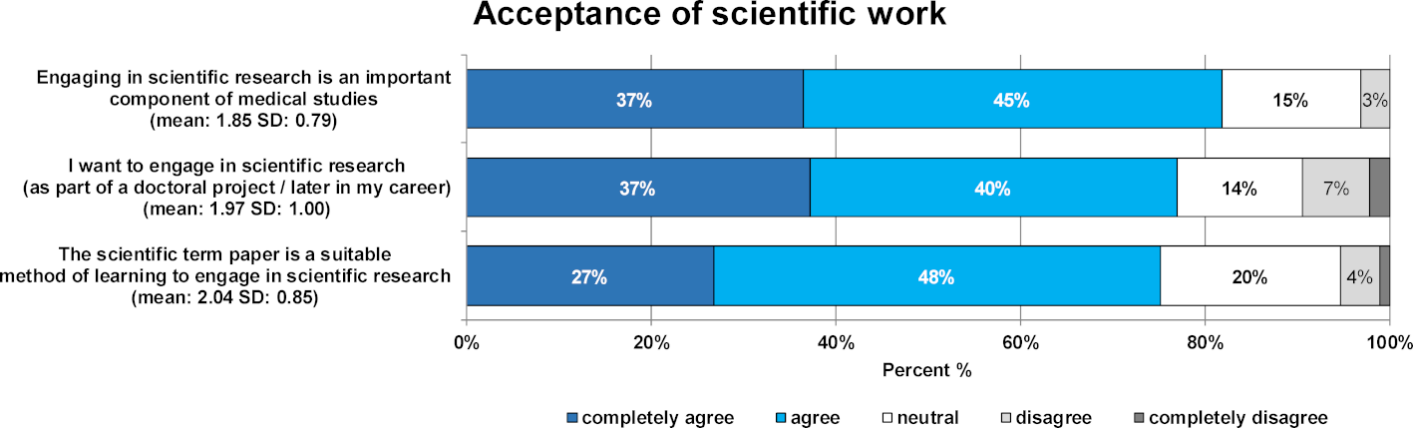
Results of the student evaluation of the acceptance of scientific work in general and the scientific term paper in particular (2^nd^ survey). In each case, the figure shows the percentage of students who rated the given statements on a 5-point Likert scale from “completely agree” to “completely disagree”. The mean and standard deviation are given in brackets.

As illustrated in figure 5 (Fig. 5), a large majority of students see scientific work as an important component of the medical degree (82%) and would like to carry out scientific research themselves (77%).

The majority of students regards the scientific term paper as part of the modular curriculum of medicine as a suitable method of learning to work scientifically (75%).

#### 3.6. Results from subsequent module evaluations

**Figure 6:**
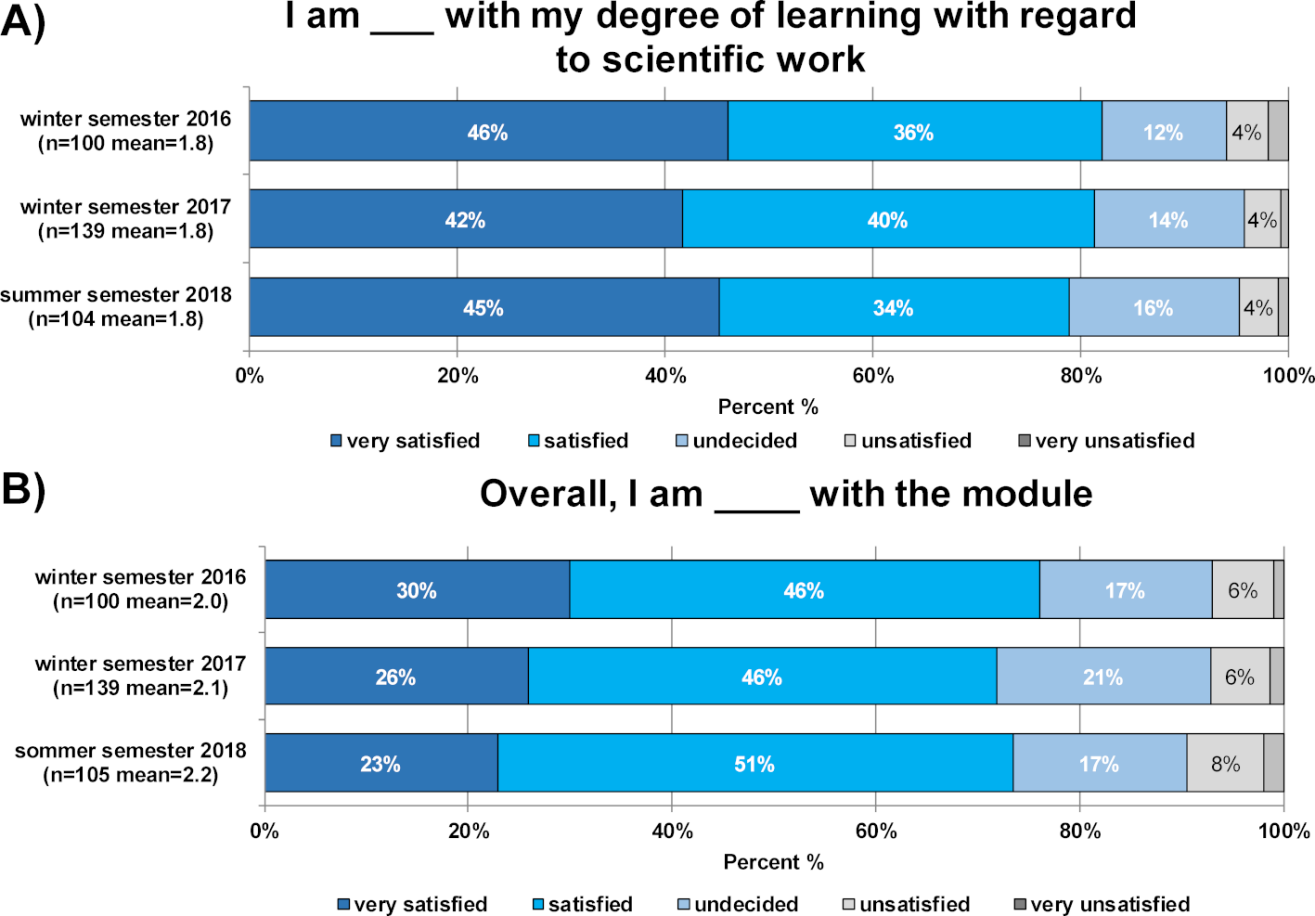
Results of the follow-up student evaluations on satisfaction with their learning growth (A) and overall satisfaction with the module (B) from the winter semesters of 2016 and 2017 and the summer semester of 2018. In each case, the figure shows the percentage of students who rated the given statements on a 5-point Likert scale from "very satisfied" to "very unsatisfied". The number of participants and the mean are given in brackets.

As shown in figure 6 (Fig. 6), the follow-up evaluations for the years 2016, 2017 and 2018 continuously show high satisfaction scores with regard to overall satisfaction with the module and satisfaction with learning growth in relation to scientific work.

As a result of the 2014 evaluation (2^nd^ survey), minimum criteria for supervision were defined. These criteria can be claimed by the students and provide guidance for the supervisors. In the follow-up evaluations for the years 2016, 2017 and 2018, the following ratings were respectively obtained: 88%, 94% and 95% for a preliminary discussion before the start of the module; 78%, 88% and 92% for an interim discussion during the module run; 48%, 42% and 37% for the trial presentation for the students’ congress; and 89%, 85% and 78% for at least one feedback on the written term paper.

## 4. Discussion

A competency-based, outcome-oriented concept for students’ own scientific research work has been developed and implemented with the scientific term paper module. The students themselves rated their acquisition of scientific competencies as high. Supervisors assessed the majority of written and oral performance assessments by students as good or very good. This finding is of particular relevance, as this proves that the majority of students have achieved the outcomes for their own scientific research activities regarding both the Charité and NKLM learning outcomes for undergraduate medical education. The student evaluation results showed that most students were satisfied with the overall concept and the quality of individual aspects of the module. Many students, however, would prefer to have more contact with their supervisor, and a small number felt insufficiently supervised. Many students would like to continue performing scientific research.

In the context of a recent increase in science modules, project works and longitudinal science tracks as part of German medical degree programmes, this report shows that a scientific term paper placed in the middle of the medical programme enjoys a high level of acceptance by students. As a very similar concept had already been established as part of the regular and reformed curricula of medicine at the Charité, many aspects seem to be transferable to other German medical faculties. Comparable modules have been introduced in, for example, Aachen, Mannheim, Köln, Heidelberg, Tübingen, Hannover, Jena and Hamburg [4], [15], [16], [17], [18], [19], [20], [21], [28] and are planned in Augsburg [29]. From our point of view, four aspects are of key importance for the transferability of the concept:

1. A central office is required, which acts as the first point of contact for students and supervisors and operationalizes guidelines for the scientific term project.
2. These guidelines communicate the horizon of expectations to everyone involved.
3. In addition, there must be faculty support for the concept, which is a prerequisite for attracting many committed supervisors and interesting topics for students.
4. Another important prerequisite for successful implementation is sufficient dedicated time for self-directed work on the project, coupled with the availability of courses and materials that guide and support students in executing their project.

It is particularly important to establish the scientific term paper as part of a larger curriculum on scientific practice, which has been achieved at the Charité in the form of 3 connected longitudinal modules. The results of the KuLM study were significant for this: students from the previous regular and reformed curriculum of medicine equally rated the competency “scientific thinking and working” as relevant for their later work as physicians, but the implementation was rated significantly better in the similarly thematically bundled structure of the reformed programme [30].

Although students were highly satisfied with various aspects of module 23 in the modular curriculum of medicine, it is also important to note that approximately 20% of students were unsatisfied with the supervision they received, and 30-40% wished to have closer supervision. One of the main reasons for this finding is their relative lack of practical experience and independence in dealing with scientific research questions at this point in the course. These deficits are obviously not adequately addressed by the supervisors. Investigations of other curricular concepts aimed at developing scientific competencies have shown that students often feel insufficiently trained in the areas of practical methods, statistics and literature research, as well as the critical analysis of research results [31], [32], [33]. This judgement is supported by graduate survey data recently published by Epstein et al. This finding revealed that these skills were not acquired until students were pursuing doctoral studies. Preparation during the undergraduate degree was generally viewed as insufficient [5].

Our study revealed a high level of student motivation to conduct further scientific research, despite the deficits cited above, together with strong acceptance of the scientific term paper concept. These results concur with results published on the motivation to engage in scientific research among students in the 5th year of study from both precursor curricula [34]. Other studies show comparable results [35] and indicate that other didactic concepts similar to the Charité scientific term paper module in the MCM promote a positive attitude among students towards research generally and motivate them to conduct further scientific research [36][, [37]. This finding is consistent with observations from a national survey of medical students by Ratte et al., in which the majority of participants felt that scientific competencies were highly important for their subsequent medical activity and were in favour of the implementation of a project module or scientific term paper in medical education [38].

In addition to the high motivation of the students, the good assessment results should also be emphasized. The good results were probably due to the previously communicated checklists for grading. They serve as transparent quality standards and guidance to the students in terms of both the form and content of their scientific term paper and presentation. Additionally, one should not overestimate the scope of the scientific term paper, the focus is on the process, the documentation and reflection on the scientific approach. Finally, the grading of the content by the supervisor has to be discussed critically, as it can be assumed that they also evaluate their own contribution to the choice of the topic and supervision, and a positive bias is thus possible.

Based on our survey results, various measures for the further development of module 23 were set in motion. The information material for students and supervisors has been improved so that information is easier to find and is presented more clearly. A “navigator” for students has been created to make it easier for them to follow guidelines and adhere to deadlines. Elements of the two questionnaires used were also integrated into the ongoing evaluation of the module in order to evaluate the success of these measures. For quality assurance purposes, minimum criteria for supervising scientific term paper projects were defined. The follow-up evaluations for the years 2016-2018 show that these criteria were achieved for the vast majority of students. An exception is the performance of a trial presentation, which is sometimes not even considered necessary by students. One possible obstacle here is that such a trial presentation usually occurs only when it can be easily integrated into an existing format, such as a working group meeting.

This study has limitations. It describes a curricular concept that was designed for one faculty and that has improved incrementally over many years. There is limited generalizability to other contexts. Furthermore, this evaluation was restricted to quantitative aspects, and the supervisor perspective was not investigated.

## 5. Conclusion

The module “Scientific term paper” in the modular curriculum of medicine at the Charité shows a high level of acceptance and good performance by students. The underlying concept has proven to be successful for students conducting their own scientific research as part of the medical degree programme. Further studies are necessary to determine the long-term effects of the module on students’ acquisition of competencies, their motivation for scientific research, and the quality and scope of further scientific research.

## Acknowledgements

We would like to thank all members of the module planning group and the module board members for M23 “Scientific thinking and working II” who planned and continued to develop the module. We also wish to thank the students who participated in this survey.

## Competing interests

The authors declare that they have no competing interests. 

## Supplementary Material

attachment 1

## Figures and Tables

**Table 1:**
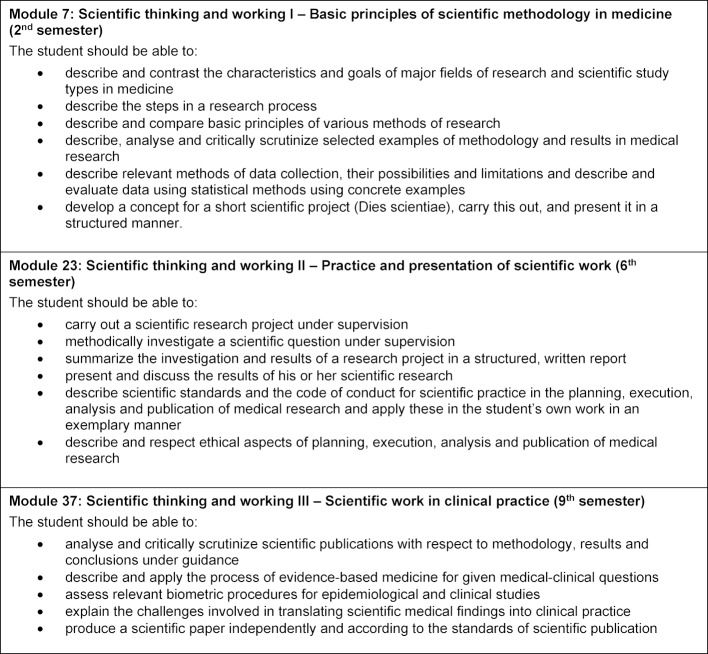
Overview of the longitudinal science curriculum for the model medical curriculum at the Charité

**Table 2:**
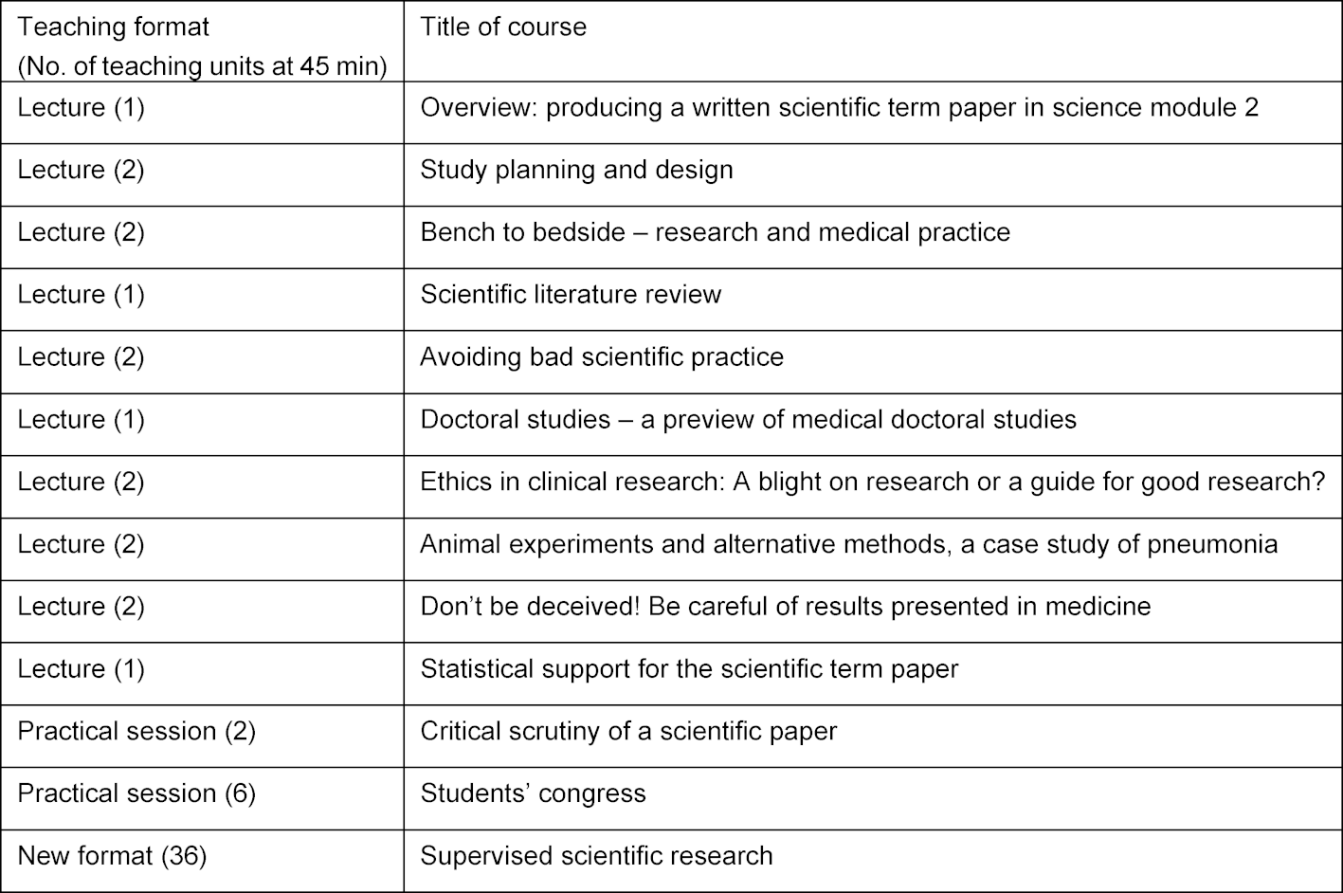
Courses for module 23 “Scientific thinking and working II” on the model medical curriculum at the Charité

**Table 3:**
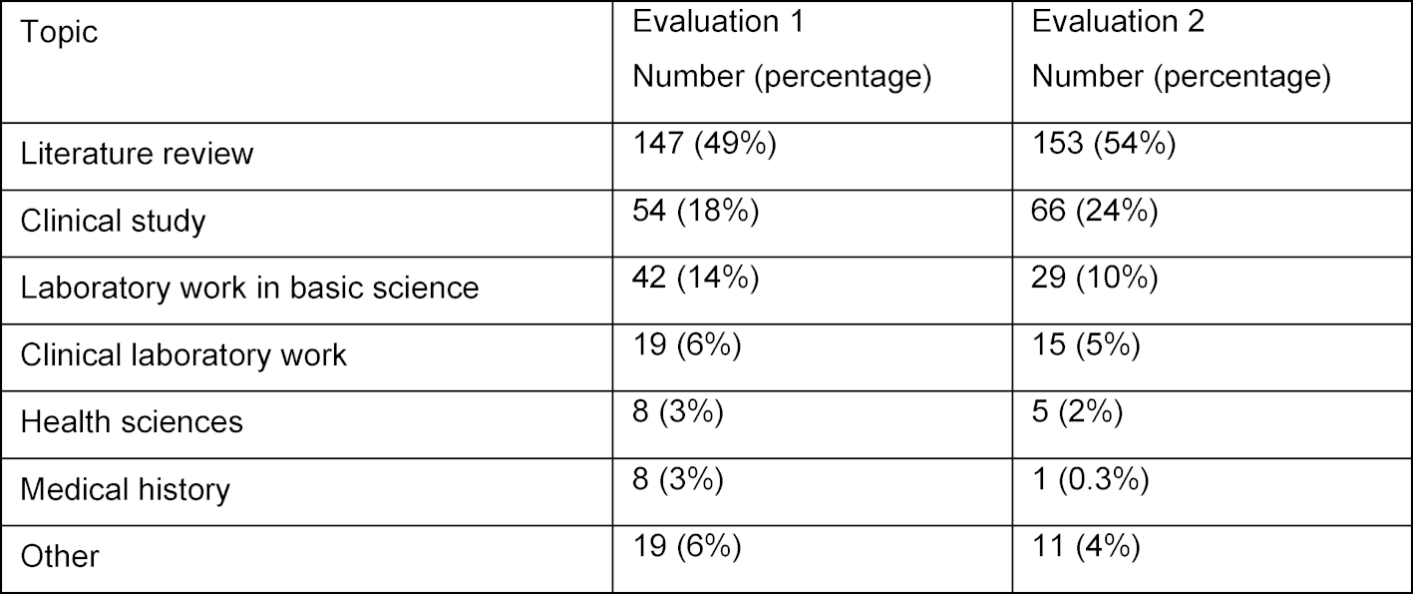
Distribution of topics for the scientific term paper
