# Supplementary material for: The scientific term paper at the Charité: a project report on concept, implementation, and students' evaluation and learning
Source: GMS J Med Educ. 2019 Oct 15;36(5):Doc53. doi: 10.3205/zma001261 (PMC6883243; doi:10.3205/zma001261)
Supplement: attachment 1 [file JME-35-53-s-001.pdf]

Attachment

1. Checklist for the formal grading of the scientific term paper (page 2)
2. Checklist for grading the content of the scientific term paper (page 4)
3. Checklist for grading the presentation as part of the students' congress (page 7)
4. Survey winter semester 2013 (page 8)
5. Survey winter semester 2014 (page 9)

Checklist for the formal grading of the scientific term paper in module 23 by the department "scientific term paper"

Student name ____________________

Student number ____________________

Supervisor ____________________

Staff member ____________________

Date ____________________

Are the formal criteria according to the guidelines for the formal structure of the scientific term paper fulfilled?

No.	Item	Fulfilled	Partly fulfilled	Not fulfilled	n/a
**1**	Was the word count specified?				
**2**	Was the word limit adhered to?				
**3**	Title page				
**4**	Table of contents				
**5**	Abstract				
**6**	Introduction				
**7**	Material and methods				
**8**	Were statistical methods described?				
**9**	Results				
**10**	Discussion				
**11**	Bibliography				
**12**	Declaration under oath				
**13**	Were the tables labelled correctly?				
**14**	Were the figures labelled correctly?				
**15**	Were references cited correctly?				
**16**	Were the formatting guidelines adhered to?				
**17**	Were the legal requirements (vote of ethics committee, animal protection, etc.) observed?				
**18**	Was a declaration made on observing the Charité "statute for protecting good scientific practice"?				
	**Sum of assessments**				

Checklist for the formal grading of the scientific term paper in module 23 by the department “scientific term paper” (continued)

Evaluation grid

> 2 times not fulfilled	failed (absolute CUT)

Term paper passed (formal) ☐

Term paper failed (formal) ☐

Points: Grade:

…………………………………………………………………………………..…………………………

Signature & Stamp, department “scientific term paper“

Checklist for grading the content of the scientific term paper in module 23

Student Surname, First name:

Student number:
1. Examiner (Supervisor):
2. Examiner (Formal grading):
Title of the term paper:

The topic of the scientific term paper was…

☐...proposed by the supervisor as a pre-structured exposé
- ☐ selected after direct contact with the supervisor
- ☐ allocated centrally after being chosen as a priority topic

☐...proposed and selected by the student
- ☐ as part of a doctoral project
- ☐ as part of a student assistant job
- ☐ neither / Student was not known to the department before

Type of scientific term paper:

The term paper project supervised by me is best described by the following category (please select only one):

☐...**1. Literature review** in the sense of a systematic review (systematic review)
☐...**2. Laboratory work in the basic sciences** (including animal experiments)
☐...**3. Laboratory work with relation to patients** (e.g., with human blood or urine samples, tumour tissue)
☐...**4. Clinical trial** (Examinations of patients / study participants; including doctor's office, nursing home)
☐...**5. Medical history project**
☐...**6. Health science project (**public health, preventive medicine, health economics, medical psychology, medical sociology or related fields)
☐...**7. Other** (please specify):____

This assessment is based on a **standard** by which the relevant research question is clearly defined and can be answered with accessible literature / data collection and analysis in the limited timespan of 6 weeks. The level of difficulty of the topic takes into account the prior knowledge of **students in the 6th semester**.

Assessment criteria		5-point scale very good to not sufficient					
		Very good	good	satisfactory	sufficient	not sufficient	Score reached
**A: Content structure**							
**Structure / Logic**							
01	The central research question of the project is derived precisely.	12	10	8	6	0	
02	The introduction clarifies the problem and the objective of the work.	12	10	8	6	0	
03	The structure is logical and balanced.	12	10	8	6	0	
Stringency							
04	The content of the term paper follows a clear structure.	12	10	8	6	0	
05	The red thread of the term paper is supported by brief introductions / summaries in important areas.	6	5	4	3	0	
			**TOTAL SCORE A:**				
**B: CONTENT / KNOWLEDGE / METHODS**							
06	The comprehensibility is supported by examples, illustrations, graphics and tables.	6	5	4	3	0	
07	The task is understood; key aspects have been covered.	6	5	4	3	0	
08	The work shows that the author has dealt intensively with the object of investigation.	6	5	4	3	0	
09	The method / literature analysis used to formulate a solution is selected professionally, discussed and justified.	3	2.5	2	1.5	0	
Ability to problematize and discuss							
10	Literature, theses and hypotheses are critically analysed and interpreted.	6	5	4	3	0	
			**TOTAL SCORE B:**				
**C: LANGUAGE**							
11	Spelling rules were followed.	3	2.5	2	1	0	
12	The language, citations and footnotes correspond to the rules of scientific research.	3	2.5	2	1.5	0	
13	The author shows solid knowledge in dealing with specialist medical terminology.	3	2.5	2	1.5	0	
			**TOTAL SCORE C:**				
**D: LITERATURE**							
14	The selection of literature shows a sufficient overview and a careful familiarization with the subject.	6	5	4	3	0	
15	Relevant primary sources were used.	3	2.5	2	1.5	0	
16	Current and international publications have been considered.	3	2.5	2	1.5	0	
			**TOTAL SCORE D:**				
**E: TIME MANAGEMENT**							
17	Agreed-upon dates and deadlines were met.	6	5	4	3	0	
18	Difficulties in implementation were adequately communicated.	3	2.5	2	1.5	0	
			**TOTAL SCORE E:**				
**Total Score (A+B+C+D+E)**							

Grading of content:

Score	Grade
>100	Very good
>85	Good
>70	Satisfactory
>55	Sufficent
<=55	Not sufficient, failed

The scientific term paper was passed / failed

(please cross out non-applicable)

And receives the grade (please spell out):

The term paper has special features / services (optional):

Name und title 1. Examiner: ____

Please indicate discipline if not physician:__________________________

Name und title 2. Examiner: ____

___ ____

City, date Signature 1. Supervisor Department stamp

The student will be …… in our working group

☐ work as a student assistant
☐ work as a doctoral student
☐ neither of both options

Please do…
1. Fill out
2. Sign and stamp
3. Send **all 3 pages** per inhouse mail to Günter Grohmann, Dieter Scheffner Fachzentrum, Wissenschaftliches Arbeiten, CCM

Please give the graded scientific term paper with copies of the grading checklist back tot he satudent directly.

Individual evaluation of the examination by a scientific staff member
Presentation of the scientific term paper in module 23 at the students' congress

The student:
First name:
Surname:
Student number:

Please use the scale of 5-1 to assess the individual aspects of the presentation

**Formal criteria**	Very good	good	satisfactory	sufficient	failed
Structure of the presentation	5	4	3	2	1
Graphical design of the presentation	5	4	3	2	1
Presentation style (Language, clarity, commitment…)	5	4	3	2	1
Adherence to the time limit	5	4	3	2	1
**Content criteria**					
Introduction to the topic	5	4	3	2	1
Clear research question (goal)	5	4	3	2	1
Description of methods	5	4	3	2	1
Presentation of results (tables / figures)	5	4	3	2	1
Presentation of limitations	5	4	3	2	1
Clear conclusions	5	4	3	2	1
Reaction to questions and contributions to the discussion	5	4	3	2	1
**What was particularly good?**					
**What could be improved?** Please formulate constructively and not hurtfully! (if necessary use reverse side)					

Passed: Yes (≥ 22 points) / No (<22 points)

Points: ____ Grade: ____

Scientific staff member:
Date:
Signature:
Name / Department (stamp where applicable):

**Points**	**Grade**
**55-48**	**Very good**
**47-40**	**Good**
**39-31**	**Satisfactory**
**30-22**	**Sufficient**
**< 22**	**Failed**

Please hand in this form together with the transcript of the examination.

Survey for students after submission of the module 23 term paper in the winter semester 2013

How did you get your scientific term paper topic?

- Searched independently
- Database of topics
- Assigned

Which type of scientific term paper did you work on?

- Literature review
- experimental
- other: ___________

How did the supervision of the scientific term paper project work?

- Individual supervision
- Working group
- other: ___________

n.a.= not applicable

**Support**	1 = very good 6 = very bad	**1**	**2**	**3**	**4**	**5**	**6**	**n.a.**
The support by the department „scientific term paper" was								
The information on Campusnet was								
The organisation of the topic selection was								
**Topics**	1 = very good 6 = very bad	**1**	**2**	**3**	**4**	**5**	**6**	**n.a.**
The breadth of topics was								
My topic was								
The support by the learning events was								
**Motivation**	1 = very high 6 = very low	**1**	**2**	**3**	**4**	**5**	**6**	**n.a.**
My motivation for scientific work before the module was								
My motivation for scientific work after the module is								
I value a scientific term paper as an assessment form …								
**Supervision**	1 = very good 6 = very bad	**1**	**2**	**3**	**4**	**5**	**6**	**n.a.**
The commitment of my supervisor was…								
The guidance by the supervisor was…								
The avaiability oft he supervisor was…								
The approximate time spent by the supervisor was _____h								
**Own work effort**	1 = very high 6 = very low	**1**	**2**	**3**	**4**	**5**	**6**	**n.a.**
My commitment to this scientific term paper was								
My diligence in documenting data was								
My diligence in scientific writing was								
My preparation for the presentation was								
I consider my learning growth due to the module to be								
My time expenditure for the scientific term paper was approximately ___h								
**Overall rating**	1 = very good 6 = very bad	**1**	**2**	**3**	**4**	**5**	**6**	**n.a.**
My proficiency in analysing relevant sources is								
My proficiency in conducting a literature search on a topic is								
I think the task of writing a scientific paper is								
The goal of M23 is to gain insight into scientific work.								
In this sense, the presentation is a sensible conclusion								
In this sense, the written scientific term paper is a sensible conclusion								

Room for free text (if necessary, use reverse side):

I am

- Female
- Male

Thank you for your participation

Survey winter semester 2014

1. **General data on the student and his / her scientific term paper**
   1.1. How did you get to your scientific term paper topic?
   - o Searched independently
   - o Database of topics
   - o Assigned

   1.2. Type of scientific term paper
   - o Literature review
   - o Laboratory work in basic sciences
   - o Laboratory work with relation to patients
   - o Clinical trial
   - o Medical history project
   - o Health Sciences project
   - o Other

   1.3. If you selected „other“, please elaborate: (Free text)

   1.4. Did you start a doctoral project before M23?
   - o yes
   - o no

   To what extent do you agree with the following statements?
   (fully agree, agree, neutral, disagree, fully disagree)

   1.5. Scientific work is an important component of medical studies

   1.6. I want to work scientifically (as part of a doctoral project / later in my career)

   1.7. The scientific term paper is a suitable method of learning to work scientifically

2. **Preparation**
   2.1. I informed myself of the scope and requirements of module 23 before the beginning of the module.
   - o yes
   - o no

   2.2. I felt informed about my supervisor’s expectations
   - o yes
   - o no

   2.3. I talked to my supervisor about communicating with him / her and the work processes during the creation the scientific term paper
   - o yes, before the beginning of the module
   - o yes, right at the beginning of the module
   - o no, these aspects were not talked about

   2.4. Before the start of the module I had contact with my supervisor by mail or telephone
   - o yes
   - o no

   2.5. I personally met with my supervisor before the housework started
   - o yes
   - o no

3. **Creation of the scientific term paper**
   3.1. How many times did you personally meet with your supervisor while preparing the scientific term paper?
   - o never
   - o 1-2x
   - o 3-4x
   - o 5-6x
   - o 7x or more

   3.2. 3.2. How often did you have contact with him / her during the preparation of the term paper by email or phone?
   - o never
   - o 1-2x
   - o 3-4x
   - o 5-6x
   - o 7x or more

   3.3. I would have liked ____ contact with my supervisor during the preparation of the term paper
   (much more, more, the same amount, less, much less

   3.4. The support in the preparation of the scientific term paper by my supervisor was
   (Very bad, bad, sufficient, good, very good)

   3.5. He / she made suggestions for changes to ____ draft(s) of my scientific term paper
   (no; one; two; three; four or more)

   3.6. He / she responded to inquiries promptly
   - o yes
   - o no

   3.7. I would have liked ____ feedback from the supervisor on the preparation of the term paper

(much more, more, the same amount, less, much less)

4. Students' congress

4.1. I discussed the presentation for the students' congress together with my supervisor

o yes o no

4.2. I presented my presentation before the students' congress in the working group of my supervising institution

o yes o no

4.3. I would have liked ____ feedback from the supervisor on the preparation of the presentation for the students' congress

(much more, more, the same amount, less, much less)

5. Satisfaction

5.1. Overall, I felt well supervised

(fully disagree, disagree, neutral, agree, fully agree)

5.2. My supervisor should supervise scientific term papers in the future

o yes o no

5.3. I experienced a ___ growth in learning with regard to scientific work in module 23

(very low, low, big, very big)

o I plan to start a doctoral project with my supervisor

o yes o no o maybe

5.4. General remarks, other comments (*free text)*
